# “Quality of Life in Epidermolysis Bullosa” and “Epidermolysis Bullosa Burden of Disease”: Italian translation, cultural adaptation, and pilot testing of two disease-specific questionnaires

**DOI:** 10.1186/s13052-024-01657-2

**Published:** 2024-04-19

**Authors:** May El Hachem, Andrea Diociaiuti, Giovanna Zambruno, Tonia Samela, Francesca Ferretti, Claudia Carnevale, Renata Linertová, Christine Bodemer, Dédée F. Murrell, Damiano Abeni

**Affiliations:** 1 Dermatology Unit and Genodermatosis Research Unit, Translational Paediatrics and Clinical Genetics Research Division, Bambino Gesù Children’s Hospital, IRCCS, Piazza Sant’Onofrio 4, 00165 Rome, Italy; 2 Clinical Epidemiology Unit, IDI-IRCCS, Rome, Italy; 3 Clinical Psychology Unit, IDI-IRCCS, Rome, Italy; 4 Gastroenterology and Nutrition Unit, Bambino Gesù Children’s Hospital, IRCCS, Rome, Italy; 5 Fundación Canaria Instituto de Investigación Sanitaria de Canarias (FIISC), Las Palmas de Gran Canaria, Spain; 6 Department of Dermatology, Reference Center for Genodermatoses and Rare Skin Diseases (MAGEC), Filière Maladies Rares Dermatologiques (FIMARAD), ERN-Skin, Hôpital Universitaire Necker- Enfants Malades, Assistance Publique - Hôpitaux de Paris-Centre (AP-HP5), Paris, France; 7 Department of Dermatology, St George Hospital & University of New South Wales, Sydney, Australia

**Keywords:** Inherited epidermolysis bullosa, Epidermolysis bullosa simplex, Junctional epidermolysis bullosa, Dystrophic epidermolysis bullosa, Kindler epidermolysis bullosa, Quality of life, Family burden, Dermatology

## Abstract

**Background:**

Inherited epidermolysis bullosa (EB) is a clinically and genetically heterogeneous group of skin fragility disorders characterized by blister formation following minor trauma. Four major types are distinguished based on the level of cleavage within the skin. Most EB forms present severely disabling cutaneous and systemic signs and symptoms. Management relies on daily time-consuming and distressing topical medications, and symptomatic treatment of systemic findings. Disease manifestations, symptoms, and daily care strongly affect patient and caregiver quality of life (QoL). To date, there are two validated EB-specific questionnaires, the “Quality of Life in Epidermolysis Bullosa” (QOLEB) and the “Epidermolysis Bullosa Burden of Disease” (EB-BoD) for the evaluation of patient and family disease burden, respectively. The aim of our study was to develop an Italian translation of the two questionnaires and to pilot-test them.

**Methods:**

The guidelines for translation and cross-cultural adaptation of health-related QoL measures were followed. Initially, two separate translations were generated for each questionnaire, and subsequently reconciled by an expert committee. This was followed by a back-translation process. The original texts and all translations underwent revision by the expert committee, resulting in definitive versions. The final versions were then tested in a pilot study involving cognitive debriefing in a group of 17 families, representative of all EB major types.

**Results:**

The translation and reconciliation process led to minor changes to obtain semantic/idiomatic/cultural equivalence of the Italian versions with the original ones and to reconcile the questions with the answer options. The cognitive debriefing process showed a good understanding and did not require text modifications.

**Conclusions:**

The Italian versions of the QOLEB and EB-BoD provide valuable tools in everyday clinical practice of reference centers, and they allow the participation in multicenter international real-life observational studies as well as in controlled clinical trials. They enable the identification of disease-specific psychological and socioeconomic challenges for EB patients and their families, guiding targeted interventions to ensure appropriate and timely care.

## Background

**Fig. 1:**
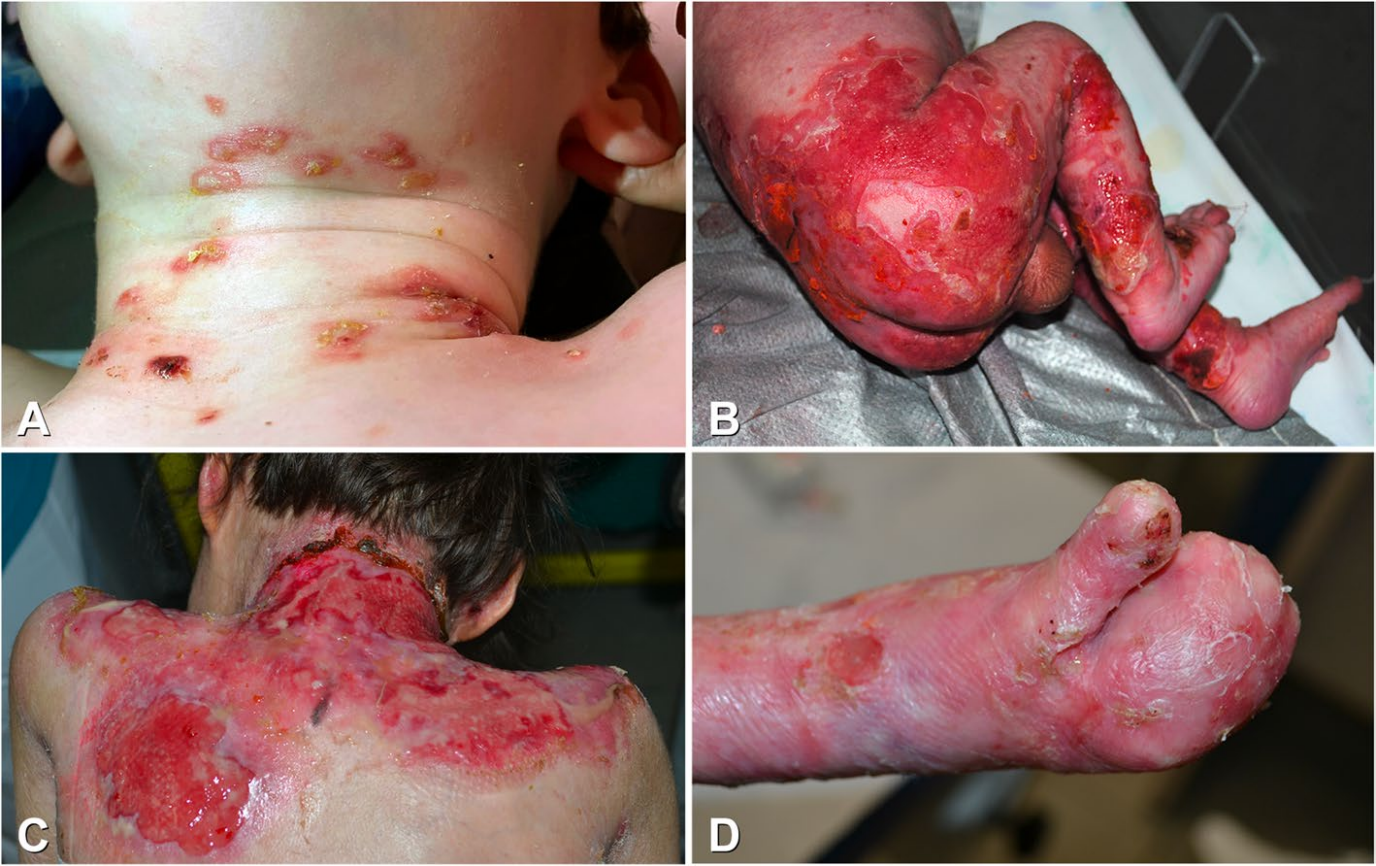
Clinical features of cutaneous manifestations of epidermolysis bullosa (EB). Serous blisters on erythematous skin in an arcuate distribution on the neck of a 4-year-old male affected with severe EB simplex (**a**); extensive erosions and peripheral blisters on the lower back, buttocks and lower limbs of a two-day-old newborn with severe junctional EB, these lesions are painful and at high risk of infection (**b**); extensive chronic and painful wound with exuberant granulation tissue, partially surrounded by crusts, on the nape and upper back of a 9-year-old girl affected with severe recessive dystrophic EB (**c**); disabling mitten hand deformity, typical of recessive dystrophic EB in an adult women (**d**)

Inherited epidermolysis bullosa (EB) comprises a clinically and genetically heterogeneous group of rare fragility disorders of the skin and mucous membranes due to defects in protein components mediating epithelial adhesion [1, 2]. EB manifests with blister formation after minor trauma, more frequently at birth or in the first days of life (Fig. 1a, b). Four major types of EB are distinguished based on the level of blister formation: EB simplex (EBS), junctional EB (JEB), dystrophic EB (DEB), and Kindler EB (KEB) [1, 2]. In addition, more than 30 subtypes with an extremely wide range of clinical features and severity are recognized, including syndromic variants. Depending on the level of skin cleavage, blister rupture results in superficial erosions or wounds, the latter frequently becoming chronic overtime and healing with scarring (Fig. 1b, c). In addition, blisters can involve mucous membranes, primarily the oral cavity, but also the esophagus, anus, eye, upper respiratory tract, and genito-urinary mucosa [2, 3]. Disease complications include recurrent infections, chronic anemia, malnutrition, failure to thrive and growth delay, hand and foot mitten deformities (Fig. 1d), joint flexion contractures, microstomia and ankyloglossia, esophageal and anal strictures. Furthermore, osteopenia and osteoporosis, delayed puberty, cardiomyopathy, renal failure, and increased susceptibility to aggressive skin squamous cell carcinomas may be present [2–4]. Thus, some EB forms are associated with a reduced life expectancy, while others are even early lethal [1, 2]. Among EB symptoms, wound-related acute and chronic pain is particularly severe and debilitating [2–4]. Moreover, chronic itching is a frequent complain, it can affect both wounds and intact skin resulting in an itch–scratch vicious cycle with further skin lesion worsening [2–4].

Disease manifestations, symptoms and complications strongly affect quality of life (QoL) of the patients and their families. As no curative treatment is yet available, EB management relies on symptomatic measures, including wound care, nutritional support, gastrostomy, esophageal dilation, hand surgery, and squamous cell carcinoma treatment [2–4]. Importantly, wound care is a crucial aspect of EB management that must be performed regularly: it is highly painful and time-consuming, thus feared by patients and distressing for their caregivers [3, 4]. In addition, many patients require frequent follow-up visits and hospitalization in reference centers, which are not always close to their residence, with financial implications and missed school/work days for patients and caregivers [5]. Finally, EB manifestations alter the physical appearance and affect the self-perception of patients [5].

The impact of the disease and patient care on QoL has been at first evaluated by specialty-specific questionnaires, in particular the Skindex-29, the Dermatology Life Quality Index (DLQI) and the Family Dermatology Life Quality Index (FDLQI) [6–10]. More recently, disease-specific instruments have been developed to assess more precisely the impact and consequences of the different EB types [11, 12]. Specifically, the “Quality of Life in Epidermolysis bullosa” (QOLEB) aims to accurately evaluate functional and emotional aspects of QoL in EB patients [11]. It has been translated and validated in several languages including Mexican Spanish, Dutch, Brazilian-Portuguese, Castilian Spanish, Farsi, and Hindi [13–20]. The second questionnaire, “Epidermolysis bullosa Burden of Disease” (EB-BoD) is intended to appraise family disease burden, in particular aspects concerning family and child’s life, disease and treatment, as well as social impact [12]. It has been developed and validated in French, and then translated into English.

The aim of our study was to develop an Italian translation of the English and French original versions of the QOLEB and EB-BoD questionnaires, respectively, and to pilot test them in a group of patients and caregivers, representative of all EB types.

## Methods

### Original questionnaires

The QOLEB is a self-administered questionnaire conceived for all EB types, which comprises 17 items addressing two broad domains: functioning and emotions [11].

Answer options are question-specific, listed from the least to the most impacting on QoL, and scored from 0 to 3; thus, higher scores indicate a greater impact. The developers of the questionnaire reported that EB patients from the age of 11 years were able to complete the QOLEB without parental assistance [11]. The QOLEB has been shown to discriminate between different EB types and severity scores evaluated using the “Epidermolysis Bullosa Disease Activity and Scarring Index” (EBDASI) and the “Birmingham Epidermolysis Bullosa severity score” (BEBs), and to correlate with DLQI, the Stanford Health Assessment Questionnaire for Mobility, and the Hospital Anxiety and Depression Scale [11, 19].

The EB-BoD questionnaire was originally developed in French. It is also self-administered and comprises 20 items addressing four domains: family life, child’s life, disease and treatment, and economic and social impact [12]. Answers are given on a 6-point Likert scale: never, rarely, sometimes, often, very often, constantly; not applicable is also included. Higher scores indicate a greater family burden of EB. The EB-BoD score negatively correlates with the mental component score of the Short Form-12 Questionnaire.

### Translation

The Ethical Committee of the Bambino Gesù Children’s Hospital (OPBG) approved the study of the Italian translation, cultural adaptation, and pilot testing of the QOLEB and EB-EB-BoD questionnaires. The guidelines for cross-cultural adaptation of health-related QoL measures were followed [21]. For each questionnaire, a forward translation was produced independently by two native Italian speakers, one being a dermatologist expert in EB, and then underwent a reconciliation step by an expert committee, according to the following criteria: the translation should reflect the original English and French texts, respectively, and Italian culture must be taken into account in choosing the words and constructing the sentences. The draft Italian text of the QOLEB was then back-translated by an English mother tongue speaker and a dermatologist expert in the disease and fluent in English. A French mother tongue speaker and a dermatologist expert in EB and fluent in French followed the same procedure for EB-BoD questionnaire. Then, the expert committee further revised the original text and all translations, evaluating equivalence between the source and the translated questionnaires in the areas of semantic, idiomatic, experiential, and conceptual equivalence. The pre-pilot testing version was submitted to the developers of both questionnaires for approval together with an interim report.

### Pilot testing

Following approval by the QOLEB and EB-BoD developers (DFM and CB, respectively), pilot testing of the Italian translated version of QOLEB was performed by cognitive debriefing on 10 patients who gave written informed assent or consent, depending on their age. Pilot testing of the Italian translated version of EB-BoD was performed by cognitive debriefing on 12 caregivers who gave written informed consent.

The participants were recruited from families, with at least one child affected with EB, attending the Reference Centre for Rare Skin Diseases of OPBG. A dermatologist contacted patients and parents, explained the aims of the project, and enrolled patients and parents who gave their written informed consent. Patients and parents completed the questionnaires on their own. A cognitive debriefing form was also administered where, for each question, they were asked to rephrase the sentence and to specify if they found the question difficult to understand or unclear. After the questionnaires were completed the participants were interviewed by the dermatologist, who enquired about the questions flagged as problematic. The interviewer took notes of the family comments on the cognitive debriefing standardized form.

### Data analysis

The expert committee reviewed the results of all interviews, prepared a summary of participants’ comments, and made decisions on questions identified as problematic. The final versions of the questionnaires were submitted to the authors of the English QOLEB and French EB-BoD for final approval.

## Results

The forward translation of the questionnaires was performed independently by an experienced dermatologist (GZ for QOLEB, and MEH for EB-BoD) and a professional translator. The translations were evaluated for reconciliation by the expert committee, which comprised an epidemiologist (DA), 4 dermatologists (AD, CC, GZ, MEH), a psychologist (TS), and language professionals.

Among the 17 items of the QOLEB, complete agreement was observed in five items between translators, aligning with the original English version. However, for the remaining 12 items, minor discrepancies in wording emerged between the translations. In particular, for 10 of these items (nine questions and one answer), there existed comparable meaning between the translations. The expert committee opted to prioritize wording that closely mirrored the original questionnaire while ensuring clarity and comprehensibility in both questions and answers. For questions with quantitative answer options (e.g., not at all, a little, a lot, etc.), the generic English term “How”, has been translated more specifically with “How much” or “To what extent”. Notably, in five of these 12 items, the translators chose a different Italian wording to achieve semantic, idiomatic, or cultural equivalence. The details about the reconciliation for these five items are shown in Table 1.

**Table 1:** Concerns and comments from expert committee explaining wording modifications in the Italian version of the “Quality of Life in Epidermolysis Bullosa” (QOLEB) and “Epidermolysis Bullosa Burden of Disease” (EB-BoD) questionnaires

QOLEB questionnaire		
**Question N**	**Concerns**	**Discussion and final choice**
5	Last answer option: in Italy, the term “nutrition” is mostly used by healthcare professionals and not by lay people	It was decided to replace “nutrition” with “feed myself”, in order to ensure better understanding of the answer option
7	The expression “involvement in sports” is not really used in Italian	It was decided to replace “involvement in sports”, which sounds awkward in Italian, with “sport activities”
13	The last answer option of the question states “…restricts my social interaction”. Social interaction is a phrase not commonly used in Italian	It was decided to replace “social interaction” with “social life”, to use a more familiar phrase
15	The term “financially” is not usually used to indicate disease costs and economic consequences for families (but for companies, firms, etc.)	It was decided to replace “financially” with “economically”, which more closely reflects the use of this term in everyday life and family settings
17	The passive form “how uncomfortable are you made to feel by others…” is not used in Italian	A more direct wording: “How uncomfortable do others make you feel…” was chosen
**EB-BoD questionnaire**		
**Question N**	**Concerns**	**Discussion and final choice**
13	The expression “…the odor produced by skin disease….” is not used in Italian	“…the odor produced by skin disease….” Was replaced by “….the odor caused by….”
15	The French expression “…faire garder mon enfant…” [“…find child care…” – in the English version], as well as the English one, do not have a direct equivalent in Italian	”…faire garder mon enfant…” was replaced by “… to find a person who takes care of my child…”
19 and 20	The term “Each time” does not fit with the possible answer options [e.g., “never”, “rarely”, etc.] in both questions	It was decided to replace the term “Each time” with “When”

Following reconciliation, one English mother tongue translator and an expert dermatologist fluent in English (AD) independently back translated the Italian text. There was complete agreement between the two translators on one item, linguistic equivalence for 11 items. Specifically, in the five items mentioned above, the original meaning of questions and/or answers was preserved though using a slightly different wording, which reflected the Italian choices (see Table 1). The authors of the QOLEB approved the initial back translation. The committee then revised the original questionnaire and all translations, and evaluated equivalence between the source and the translated questionnaires. The pre-pilot version was approved by the QOLEB authors.

As to the EB-BoD instrument, there was full agreement in 13 out of 20 items between the translators and with the original French version, and in three additional items, there was meaning correspondence between both versions. The wording of the remaining four items was slightly modified to clarify the questions in relation to the different answer options (items 19 and 20) or to obtain semantic/idiomatic/cultural equivalence of the Italian version with the French one (items 13 and 15) (Table 1). Following reconciliation, one French mother tongue translator and a French mother tongue clinical expert (FF) back translated the Italian text. There was complete agreement between the two translators. The authors of the EB-BoD approved the initial back translation. The committee then followed the same procedure described above for the QOLEB, and the pre-pilot version was approved by the EB-BoD authors.

Questionnaire cognitive debriefing was performed on 17 families with at least one individual affected with EB (Table 2). All EB subtypes were represented: four EBS, three JEB, nine DEB and one KEB. Specifically, the QOLEB was administered to 10 patients aged > 11 years (1 EBS, 3 JEB, 5 DEB, and 1 KEB), and the EB-BoD to 12 parents of 11 children affected with different EB types (4 EBS, 1 JEB, 6 DEB). Interestingly, 10 out of 12 caregivers who filled the questionnaire and cognitive debriefing forms were the mothers of affected individuals, and the remaining two were the fathers. Table 2 also summarizes other information about parents: median age was 39 years (minimum 34, maximum 51), most of them were highly educated, and 11/12 were employed (one retired). All patients and all caregivers completed their respective questionnaires in 15 min or less (details in Table 3).

**Table 2:** Characteristics of the patients and caregivers involved in the pilot testing of the two disease-specific questionnaires, “Quality of Life in Epidermolysis Bullosa” and “Epidermolysis Bullosa Burden of Disease”

**Variable**	**Levels**	**Patients**			**Caregivers**	
		**N**	**%**		**N**	**%**
Sex	Male	9	52.9		2	16.7
	Female	8	47.1		10	83.3
Age (years)	0-10	7	41.2	<40	6	50.0
	11-17	4	23.5	≥40	6	50.0
	≥18	6	35.3			
		**Median**	**Min-Max**		**Median**	**Min-Max**
		14	1-56		39	34-51
Education	Primary				0	0.0
	High School				2	20.0
	University				8	80.0
	(Missing)				2	
Work	Yes	1	5.9		11	94.1
	No (Unemployed)	1	5.9			
	At home	3	17.7			
	Still at school	10	58.8			
	Retired	2	11.7		1	5.9
Diagnosis	EBS	4	23.5			
	JEB	3	17.7			
	DEB	9	52.9			
	KEB	1	5.9			

*EBS* Epidermolysis bullosa simplex, *JEB* Junctional epidermolysis bullosa, *DEB* Dystrophic epidermolysis bullosa, *KEB* Kindler epidermolysis bullosa

**Table 3:** Completion percentages and time for completion for the two study questionnaires, “Quality of Life in Epidermolysis Bullosa” and “Epidermolysis Bullosa Burden of Disease”

**Variable**	**Levels**	**Patients**		**Caregivers**	
		**N**^**a**^	**%**	**N**	**%**
Complete	Yes	10	100.0	12	100.0
	No	0	0.0	0	0.0
		**Median**	**Min–Max**	**Median**	**Min–Max**
Time	(minutes)	10	3–15	5	2–15
	^b^Missing			2	

^a^Seven patients were below age 11, and were not administered the “Quality of Life in Epidermolysis Bullosa” questionnaire

^b^Questionnaires completed, but lacking information about the time needed to respond

During the cognitive debriefing, most respondents demonstrated a clear understanding of both questionnaires, except for an issue raised regarding the last answer to question five in the QOLEB questionnaire: "I rely on my gastrostomy tube for nutrition." Specifically, one adult patient, affected by an intermediate form of JEB, did not know the term "gastrostomy," and thus did not understand the answer. Additionally, regarding the QOLEB questionnaire, there was a suggestion to add “when” to questions 8 and 15, thus becoming “How and when…”. The rationale behind this was to account for occasional feelings of frustration and depression, emphasizing the relevance of a temporal aspect in both questions. While intriguing, this suggestion was deemed to significantly alter the original question, falling outside the intended scope of the questionnaire translation and cross-cultural validation. Consequently, it was not integrated into the questionnaire.

Regarding EB-BoD question 9, "My family does not come to see us because of my child’s skin disease," a parent proposed a positive rephrasing: "My family comes to see us despite my child’s skin disease." Similarly, there was a suggestion to positively modify question 15 from “I have great difficulty in finding child care for my child on account of his/her skin disease” to “I easily find child care for my child despite his/her skin disease.” However, both proposed changes couldn't be accepted due to their substantial alteration of the original meaning and text, which would also impact the scoring system.

Overall, the expert committee did not modify the Italian version of the two questionnaires following cognitive debriefing. The validated Italian texts (Tables 4. and 5) were forwarded again to the respective developers for final approval.

**Table 4.**
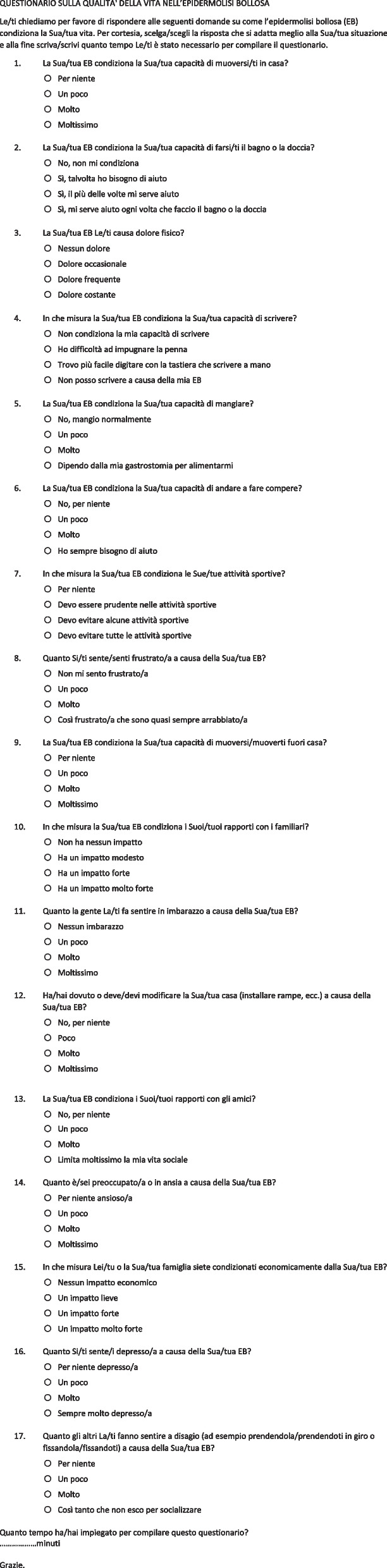
Italian version of the Quality of Life in Epidermolysis Bullosa questionnaire^a^

^a ^The copyright of this questionnaire belongs to the Australasian Blistering Diseases Foundation

**Table 5:** Italian version of the Epidermolysis Bullosa Burden of Disease questionnaire

La malattia di Suo/a figlio/a è oggi ben conosciuta. Tuttavia, l’impatto e le conseguenze di questa malattia sulla Sua vita quotidiana sono meno conosciuti Per ognuna delle seguenti affermazioni, può scegliere tra sette risposte possibili. Non ci sono risposte giuste o sbagliate. Per cortesia, risponda nella maniera più spontanea possibile pensando alla Sua situazione nelle ultime 4 settimane								
		Sempre	Molto spesso	Spesso	Qualche volta	Raramente	Mai	Non mi riguarda
1	La malattia della pelle di mio/a figlio/a ci ha spinto a volerci trasferire							
2	La malattia della pelle di mio/a figlio/a mi ha portato a voler lasciare Il mio lavoro							
3	Penso alla malattia della pelle di mio/a figlio/a tutto il giorno							
4	Cerco di proteggere mio/a figlio/a a causa della sua malattia della pelle							
5	La malattia della pelle di mio/a figlio/a ci impedisce di andare in vacanza							
6	Mio/a figlio/a ha bisogno di più attenzione degli altri a causa della sua malattia della pelle							
7	La malattia della pelle di mio/a figlio/a ci ha costretti a rimettere in discussione i nostri progetti per il futuro							
8	La malattia della pelle di mio/a figlio/a mi impedisce di andare a trovare la mia famiglia							
9	La mia famiglia non viene a trovarci a causa della malattia della pelle di mio/a figlio/a							
10	La malattia della pelle di mio/a figlio/a ci crea problemi di coppia							
11	Le visite mediche per la malattia della pelle di mio/a figlio/a mi causano spesso un senso di frustrazione							
12	Le reazioni della gente di fronte alla malattia della pelle di mio/a figlio/a sono difficili da accettare							
13	Faccio fatica ad accettare la malattia della pelle di mio/a figlio/a							
14	Faccio fatica ad abituarmi all’odore causato dalla malattia della pelle di mio/a figlio/a							
15	Ho grandi difficoltà a trovare una persona che si prende cura di mio/a figlio/a a causa della sua malattia della pelle							
16	Mio/a figlio/a ha grandi difficoltà a scuola a causa della sua malattia della pelle							
17	Ho paura per il futuro di mio/a figlio/a a causa della sua malattia della pelle							
18	Le cure necessarie a mio/a figlio/a iniziano a pesarmi							
19	Quando devo andare in ospedale, il giorno prima non mi sento bene							
20	Quando vado in ospedale, il giorno dopo non mi sento bene							

## Discussion

To date, no disease-specific validated questionnaires for the measure of QoL and family disease burden for EB are available in Italian. Indeed, previous studies on QoL and family impact in EB have employed generic instruments, such as the Short Form-36 and the General Health Questionnaire-12, as well as dermatology-specific questionnaires: Skindex-29, DLQI and its version for children (CDLQI), and FDLQI [6–10]. Although these validated instruments offer valuable measures for comparison with other diseases, both dermatological and non-dermatological, they do not fully encompass the complexity of disease manifestations and symptoms, and consequently their impact on the QoL for patients with EB and their caregivers.

Disease-specific questionnaires translated into national languages are a relevant tool to assess QoL and socio-economic impact in different nations and across cultural backgrounds. They can be exploited, also, to evaluate QoL changes overtime and, more importantly, during multicenter international therapeutic trials [22]. Indeed, the availability of such tools is one of the aims of the European Reference Network for Rare Skin Disorders (ERN-Skin) [https://ern-skin.eu/what-is-the-ernskin/]. Rare and chronic skin diseases pose a major burden on patient and family QoL [6–10, 22–26]. Therefore, questionnaires designed to measure the impact of diseases such as EB on family daily life, education and working activities, economic load, and psychological and social effects are valuable and necessary instruments [22].

The development of the Italian version of the QOLEB presented minor adaptation issues related to semantic and linguistic differences between English and Italian languages. For the EB-BoD the process was even smoother, given the significant linguistic and cultural similarities between Italy and France.

Although we did perform cognitive debriefing on only a small sample of patients and caregivers in a single center, our population was representative of the EB disease spectrum, as all disease types were included. Moreover, the validation process has followed the guidelines for cross-cultural adaptation of health-related QoL measures [21], and a remarkable agreement between both the researchers/translators and the caregivers was registered. Ten out of twelve caregivers who evaluated the Italian version of EB-BoD were patient’s mothers, further confirming the crucial role of the mother as main informal caregiver in rare diseases [27, 28].

Finally, we plan to further validate the QOLEB and EB-BoD questionnaires, including the verification of the psychometric properties of our version, on a larger Italian patient cohort in the framework of a European online survey. This survey will be carried out in the next months, as part of an ongoing European project (“Changes in the socio-economic burden of epidermolysis bullosa in Europe” -BUR-EB) involving eight EU countries [29].

The validity and reliability of the QOLEB instrument in quantifying functional and emotional aspects in patients with various EB types has already been shown for the original version, as well as for the Dutch, Spanish, Brazilian-Portuguese, Farsi, and Hindi translations [11, 13–20, 30]. Moreover, QOLEB has been successfully employed in an online English cross-sectional survey on features and impact of EBS [31], and in a short-term prospective study on correlation between disease severity score, wound evolution, and QoL in DEB patients [32]. Concerning the EB-BoD instrument, preliminary analysis indicated that it could also discriminate between specific EB types [12].

Of note, the first two treatments for EB skin wounds have been recently approved by regulatory agencies. The first one, Oleogel-S10, is a gel containing triterpenes extracted from birch bark that proved effective and safe in accelerating healing of EB wounds and reducing pain [33]. Oleogel-S10 has been approved by the European Medicine Agency (EMA) and the Food and Drug Administration (FDA) for use in DEB and JEB patients aged > 6 months. The second treatment is a topical in-vivo gene therapy gel containing a replication defective herpes simplex virus type 1 carrying two copies of COL7A1 cDNA (Beremagene geperpavec) that showed efficacy in achieving healing of RDEB wounds and reducing pain [34]. Beremagene geperpavec has obtained approval by the FDA in RDEB patients aged > 6 months. Disease-specific questionnaires, in particular QOLEB and EB-BoD, will be useful tools to measure the impact on patient and family daily life of newly approved therapies. In addition, several multicenter international trials on different treatment approaches (i.e., pharmacological, cell- gene- and protein-therapy) are ongoing or about to start [35, 36]. Thus, the availability of validated Italian questionnaires contributes to provide meaningful patient-reported outcome measures for ongoing and future controlled clinical trials.

Ultimately, these tools can also serve as valuable assets in the everyday clinical practices of specialized centers. They enable the identification of particular psychological and socioeconomic challenges for EB patients and their families, guiding targeted interventions to ensure appropriate and timely care.

## Data Availability

QOLEB and EB-BoD translations, back translations, and filled questionnaires with comments obtained during pilot testing are available from MEH, on request.

## Abbreviations

CDLQI: Children dermatology life quality index
DLQI: Dermatology Life quality index
EB: Epidermolysis bullosa
EBS: Epidermolysis bullosa simplex
DEB: Dystrophic epidermolysis bullosa
JEB: Junctional epidermolysis bullosa
KEB: Kindler epidermolysis bullosa
FDLQI: Family dermatology life quality index
FDA: Food and drug administration
QoL: Quality of life
QOLEB questionnaire: Quality of life in Epidermolysis bullosa questionnaire
EB-BoD questionnaire: Epidermolysis bullosa burden of disease
OPBG: Bambino gesù children’s hospital
